# Metformin and incidence of age-related macular degeneration in people with diabetes: a population-based 5-year case-control study

**DOI:** 10.1136/bmjophth-2025-002339

**Published:** 2026-02-02

**Authors:** Dewi Fathin Romdhoniyyah, Ayesh Alshukri, David G Parry, Simon Harding, Nicholas A V Beare

**Affiliations:** 1 Eye and Vision Science, University of Liverpool, Liverpool, UK; 2 Department of Ophthalmology, Universitas Gadjah Mada, Yogyakarta, Indonesia; 3 Liverpool Ophthalmic Reading Centre, Liverpool University Hospitals NHS Foundation Trust, Liverpool, UK; 4 St Paul’s Eye Unit, Liverpool University Hospitals NHS Foundation Trust, Liverpool, UK

**Keywords:** Macular Degeneration, Drugs, Treatment Medical, Retina

## Abstract

**Objective:**

Metformin has been identified as a potential treatment for age-related macular degeneration (AMD). Photographic screening for diabetic retinopathy provides an opportunity to conduct a case-control study with systematic AMD grading. We aimed to investigate associations between metformin use and incidence and progression of AMD at different grades.

**Methods and analysis:**

We randomly sampled 2600 participants from 10 336 people aged ≥50 years with diabetes who attended retinopathy screening in 2011 (baseline) and were enrolled to the Individualised Screening for Diabetic Retinopathy study. 2545 of these participants had type 2 diabetes and gradable fundus photographs at baseline, which were graded using modified age related eye disease study grading. We used data including those on metformin prescription from general practitioner records. We used multivariate logistic regression to investigate associations between metformin and incidence or progression of early, intermediate and late AMD.

**Results:**

Of 2545 participants, 2089 attended and had gradable fundus images on year 5. Metformin was associated with reduced incidence of intermediate AMD by 5 years after adjusting for confounders (complete record OR 0.63, 95% CI 0.43 to 0.92, p=0.02). In univariate analysis, metformin was associated with reduced incidence of late AMD (OR 0.43, 95% CI 0.21 to 0.88, p=0.02) but this did not remain significant after adjusting for age and sex. The numbers progressing to late AMD were small. There was no association between metformin and the incidence of early AMD.

**Conclusion:**

We have found a significant association between metformin use and reduction in incidence of intermediate AMD by 37% in people with diabetes over 5 years. Previous epidemiological studies of metformin and AMD have used secondary data on AMD. In this observational study, there were baseline differences between groups, although significant findings remained after adjusting for important confounders. Given metformin’s anti-ageing therapeutic effects, the reduction in risk is plausible and warrants prospective clinical trials.

**WHAT IS ALREADY KNOWN ON THIS TOPIC**

- Metformin has been identified as a potential treatment for age-related macular degeneration (AMD). Epidemiological evidence of an association between metformin use and reduced AMD relies on unvalidated insurance, billing or general practitioner data with no severity grading of AMD. Population-based photographic screening for diabetic retinopathy provides an opportunity to grade for AMD and examine associations between metformin use and incidence of AMD at different levels.

**WHAT THIS STUDY ADDS**

- In people with diabetes, metformin use is associated with reduced incidence of intermediate AMD over 5 years after adjusting for potential confounding variables.

**HOW THIS STUDY MIGHT AFFECT RESEARCH, PRACTICE OR POLICY**

- This study supports a prospective clinical trial of metformin to reduce AMD progression.

## Introduction

 There is considerable interest in the effects of metformin, a common diabetes medication, on ageing and age-related macular degeneration (AMD).^1 2^ Repurposing drugs with beneficial effects on AMD offers a promising route to new treatment options that are likely to be readily available, cost-effective and safer for patients.^3^ AMD is a common cause of blindness in high-income countries,^4^ and treatment for advanced AMD has a high patient and financial burden with limited long-term success.^5^ A study of AMD genetics and drug effects using advanced bioinformatics has identified metformin as the best fit for a potential treatment for all stages of AMD.^6^ Metformin is cheap, readily accessible and has a favourable safety profile.^7^

Metformin is considered to have a beneficial role in ageing-related diseases due to its antioxidant,^8^,^11^ anti-inflammatory,^812^,^14^ anti-angiogenesis^15^,^17^ and calorie-restriction mimetic^18 19^ effects. Recently, metformin has been demonstrated to decrease age-related inflammation by promoting autophagy and optimising mitochondrial function.^20^

A recent systematic review and meta-analysis of studies investigating associations between metformin use and AMD included 18 observational studies and 2 683 234 people; nine case-control, seven retrospective cohort and two cross-sectional studies. It reported a significant reduction of the odds of AMD in metformin users (pooled OR=0.86, 95% CI 0.79 to 0.93, p=0.0002, I2=90%).^21^ The OR was more reduced for non-diabetics (pooled OR=0.70) in only two studies that reported this. Another meta-analysis included nine studies with nearly 1.5 million participants.^1^ It found that patients with diabetes taking metformin had a significantly lower OR of having or developing AMD (OR 0.63; 95% CI 0.46 to 0.86; p=0.004). The studies included in these meta-analyses used data collected from insurance claims, billing codes, hospital records or general practitioner (GP) records, which may not be accurate for AMD diagnosis, its absence or may be biased such as more likely to record neovascular AMD than intermediate AMD. Only one study diagnosed AMD from fundus photographs,^22^ and the others relied on routinely collected data and included either no, or only basic classification of AMD. To date, no studies investigating metformin and AMD have used data on AMD severity grade with a recognised classification scheme. All but one study has relied on secondary data on AMD collected during routine clinical care. This can be inaccurate particularly for AMD as it has a wide spectrum of diseases including asymptomatic stages and loosely used terminology of different stages and types.

The use of fundus images from a population that is systematically screened for diabetic retinopathy (DR) enables accurate grading for AMD. In Liverpool, the screened population with diabetes was recruited into a large research database which included GP and hospital data to enable a prospective randomised controlled trial of varying screening intervals, the Individualised Screening for Diabetic Retinopathy (ISDR) study. Using these data including fundus images, we aimed to investigate any association between metformin prescription and incidence or progression of AMD at its different stages.

## Materials and methods

We obtained data for this study from the ISDR dataset (http://www.isdrproject.co.uk/). ISDR was an National Institute of Health Research (NIHR) funded randomised study in Liverpool to investigate variable and individualised screening intervals within the Liverpool Diabetic Eye Screening Programme (LDESP).^23 24^ The ISDR study collected data from 2009 to 2017 consisting of routine GP and diabetic eye screening data on all people with diabetes in the LDESP who did not opt out of consent. Data included diabetes medication, but without dosages. ISDR had no data on diet or vitamin and anti-oxidant supplements as they are purchased by patients and not prescribed by GPs.

The calculation of the sample size required for a multivariate logistic regression model was calculated by n=10 k/p, where k is the number of predictor variables and p is the likelihood of an event.^25^ We wanted to be able to include up to 10 predictor variables (k=10), and estimated any AMD progression at 10% of participants over 5 years (probability, p=0.1) from the Blue Mountain Eye Study (5-year incidence of early or late AMD 9.8%).^26^ This gives a required sample of 1000. We allowed for a 30% drop-out rate for the 5-year assessment which gives a required sample of 1429. Resources allowed us to include additional participants, and as there was uncertainty for the 10% progression rate, we used a sample of 2600.

The ISDR cohort study dataset had 28 384 participants. There were 10 336 participants who were aged ≥50 years and attended the LDESP between 1 January and 31 December 2011, the baseline year for this study. We randomly selected 2600 participants from this group. We retrieved retinal images and clinical data including metformin medication record at baseline until year 5 (2016). Clinical data were collected from prescribing GPs imported into the ISDR database and subject to data cleaning. We excluded participants with ungradable fundus images at baseline (11) or type 1 diabetes mellitus () (44), resulting in 2545 participants.

This retrospective study of prospectively collected ISDR data was registered at the University of Liverpool (sponsorship number: 4874) and approved by the London Central Research Ethics Committee (reference: 19/LO/1201). Data used in this study are publicly available at the University of Liverpool Research Data Catalogue.^27^

One ophthalmology resident (DFR) graded dilated, non-stereoscopic, colour fundus photographs collected by the LDESP for AMD. We graded each eye using the Age Related Eye Disease Study (AREDS) grading system levels 1–4^28^ with modified level 4 to include non-foveal geographic atrophy (table 1). AREDS level 1 is considered no AMD, AREDS level 2 is early AMD, AREDS level 3 is intermediate AMD and AREDS level 4 is late AMD of either neovascular or atrophic form. A standard Early Treatment Diabetic Retinopathy Study (ETDRS) grid template and set of measuring circles used in AREDS were available in the viewing software, OptoMize (NEC Software Solutions, Hemel Hempstead, UK). Grading was performed in both eyes and AMD severity was based on the individual’s worse eye if different.

**Table 1:** Modified AREDS AMD grading^28^

AMD level	Defining features
1: no AMD	Drusen maximum size < circle C-0 (63 μm diameter) and total area < circle C-1 (125 μm diameter)
2: early AMD	Presence of one or more of the following: Drusen maximum size ≥ circle C-0 but < circle C-1 Drusen total area ≥ circle C-1 Retinal pigment/epithelial pigment abnormalities consistent with AMD, defined as one or more of the following in the central or inner subfields: Depigmentation present Increased pigment ≥ circle C-1 Increased pigment present and depigmentation at least questionable
3: intermediate AMD	Presence of one or more of the following: Drusen maximum size ≥ circle C-1 Drusen maximum size ≥ circle C-0 and total area > circle I-2 (0.17 disc diameter) and type is soft indistinct Drusen maximum size ≥ circle C-0 and total area > circle O-2 (0.44 disc diameter) and type is soft distinct
4: late or advanced AMD	Presence of one or more of the following: Geographic atrophy within grid but none at the centre of macula* Geographic atrophy in central subfield with at least questionable involvement of the centre of macula Evidence of neovascular AMD Fibrovascular/serous pigment epithelial detachment Serous (or haemorrhagic) sensory retinal detachment Subretinal/subretinal pigment epithelial haemorrhage Subretinal fibrous tissue (or fibrin) Treatment for neovascular AMD

* Modified from original AREDS grading by moving from level 3 to 4.

AMD, age-related macular degeneration; AREDS, Age Related Eye Disease Study.

Baseline images from 2011 and follow-up images from 2016 (±12 months from a patient’s screening due date) were graded separately. The graders took care to distinguish drusen from hard exudates using the non-stereoscopic features detailed in ETDRS Report 10 including distribution, shape and appearance.^29^ A randomly selected 20% of images were re-graded for quality assurance by a senior AMD grader (DGP) at the Liverpool Ophthalmic Reading Centre. During grading, any photos that prompted grading uncertainty were discussed with an experienced AMD specialist (NAVB). Resource constraints prevented double grading of all images. If patients attended the base hospital eye clinic (Royal Liverpool University Hospital), we used the additional data available, for example, to identify neovascular AMD.

We defined metformin use as a record of metformin prescription at baseline and at another time point during the 5-year follow-up. Incidence and progression of AMD were determined per individual, not per eye. Definition of AMD incidence in our study followed the definition used by the Blue Mountain Eye Study,^26^ that is, excluding participants already with AMD at that level and worse at baseline. Also, when defining the number at risk of developing early AMD, the participants with no AMD at baseline who developed intermediate or late AMD were excluded, and, for the number at risk of intermediate AMD, participants with no AMD or early AMD at baseline who developed late AMD were excluded. This ensures the appropriate population at risk for each level of AMD incidence.

Statistical analysis and random sampling were done using Stata (V.16.1 for Mac, StataCorp, Texas, USA). We used descriptive statistical analysis to describe the baseline characteristics of the study participants. In assessing associations with AMD incidence and progression, we performed logistic regression analysis. Univariate logistic regression analysis was first performed to select covariates to be included in multivariable logistic regression models. Age, sex, glycosylated haemoglobin (HbA1c) and the known duration of diabetes were considered potentially confounding variables and therefore always included in multivariable logistic regression. Other covariates with a p value <0.20 in the univariate logistic regression were included in the multivariable logistic regression analysis. If two variables with a p value <0.20 were highly correlated (r≥0.8), the one with the higher p value was not included.

We considered multivariable logistic regression effect sizes as statistically significant if the 95% CI did not contain a null value. We used two models of multivariable-adjusted logistic regression in our analysis. The first was a complete record analysis (ie, excluded missing data) adjusted for the presence of DR, HbA1c and known diabetes duration; the second was adjusted for the same variables but used multiple imputation to handle missing data using multivariate imputation using chained equations. Additional models adjusted for the above variables, plus the variables that in univariate logistic regression analysis had a p value of <0.2.

### Patient and public involvement

The ISDR study involved members of the public and people with diabetes in all aspects of its programme. Patients and public were not involved in this research project.

## Results

### Study participants’ demographics

Of 2545 randomly selected ISDR participants with type 2 DM, 456 had no or ungradable year 5 DR screening fundus photographs and were excluded from further analysis. Of 2089 included participants, there were 836 (40%) prescribed metformin at baseline and between 2011 and 2016, and 1253 (60%) who were not. We compared the baseline characteristics of participants with and without metformin use in table 2. Persons taking metformin were on average 1.7 years younger and had statistically significantly higher HbA1c, longer known DM duration, lower systolic blood pressure and less AMD at baseline. At baseline, 284 of all participants had early AMD, 90 had intermediate AMD and 12 had late AMD.

**Table 2:** Baseline characteristics of metformin and non-metformin users

	Total N=2089	Metformin group N=836	No metformin group N=1253	P value
Age				
Median (IQR), years	65.0 (58.5–71.9)	64.0 (57.7–70.4)	65.7 (59.0–72.5)	<0.001*
50–54 years, n (%)	246 (11.8%)	107 (12.8%)	139 (11.1%)	0.001†
55–59 years, n (%)	371 (17.8%)	170 (20.3%)	201 (16.0%)	
60–64 years, n (%)	425 (20.3%)	177 (21.2%)	248 (19.8%)	
65–69 years, n (%)	375 (18.0%)	156 (18.7%)	219 (17.5%)	
70–74 years, n (%)	348 (16.7%)	123 (14.7%)	225 (18.0%)	
75–79 years, n (%)	221 (10.6%)	80 (9.6%)	141 (11.3%)	
≥80 years, n (%)	103 (4.9%)	23 (2.8%)	80 (6.4%)	
Sex, n (%)				0.97†
Male	1216 (58.2%)	487 (58.3%)	729 (58.2%)	
Female	873 (41.8%)	349 (41.7%)	524 (41.8%)	
Ethnicity, n (%)				0.11†
Non-Caucasian	114 (6.3%)	52 (7.5%)	62 (5.6%)	
Caucasian	1693 (93.7%)	645 (92.5%)	1048 (94.4%)	
HbA1c				
Median (IQR), mmol/mol	50.0 (44.0–57.0)	51.0 (45.0–59.0)	49.0 (43.0–56.0)	<0.001*
≤48 mmol/mol, n (%)	792 (43.3%)	289 (38.0%)	503 (47.1%)	<0.001†
>48 mmol/mol, n (%)	1038 (56.7%)	472 (62.0%)	566 (52.9%)	
DM duration				
Median (IQR), years	5.0 (2.2–8.6)	5.2 (2.3–8.9)	4.8 (2.1–8.4)	0.008*
Same/lower than median, n (%)	1069 (51.2%)	407 (48.7%)	662 (52.8%)	0.063†
Higher than median, n (%)	1020 (48.8%)	429 (51.3%)	591 (47.2%)	
Diastolic BP				
Median (IQR), mm Hg	76.0 (70.0–80.0)	76.0 (70.0–80.0)	76.0 (70.0–81.0)	0.99*
<80 mm Hg, n (%)	1196 (63.0%)	497 (63.3%)	699 (62.8%)	0.82†
≥80 mm Hg, n (%)	702 (37.0%)	288 (36.7%)	414 (37.2%)	
Systolic BP				
Median (IQR), mm Hg	133.0 (124.0–140.0)	132.0 (124.0–140.0)	134.0 (126.0–140.0)	0.004*
<130 mm Hg, n (%)	684 (36.0%)	312 (39.7%)	372 (33.4%)	0.005†
≥130 mm Hg, n (%)	1216 (64.0%)	474 (60.3%)	742 (66.6%)	
Total cholesterol				
Median (IQR), mmol/L	4.0 (3.5–4.8)	4.1 (3.6–4.7)	4.0 (3.5–4.8)	0.74*
<4 mmol/L, n (%)	795 (45.3%)	334 (45.3%)	461 (45.3%)	0.98†
≥4 mmol/L, n (%)	960 (54.7%)	404 (54.7%)	556 (54.7%)	
HDL				
Median (IQR), mmol/L	1.2 (1.0–1.4)	1.2 (1.0–1.4)	1.2 (1.0–1.4)	0.05*
≥1 mmol/L, n (%)	1474 (84.6%)	602 (82.6%)	872 (86.0%)	0.05†
<1 mmol/L, n (%)	269 (15.4%)	127 (17.4%)	142 (14.0%)	
LDL				
Median (IQR), mmol/L	2.0 (1.5–2.6)	2.0 (1.6–2.6)	2.0 (1.5–2.6)	0.11*
<2 mmol/L, n (%)	803 (47.6%)	322 (45.3%)	481 (49.3%)	0.10†
≥2 mmol/L, n (%)	884 (52.4%)	389 (54.7%)	495 (50.7%)	
Triglycerides				
Median (IQR), mmol/L	1.6 (1.1–2.1)	1.5 (1.1–2.1)	1.6 (1.1–2.1)	0.97*
≤1.7 mmol/L, n (%)	1001 (59.9%)	426 (60.4%)	575 (59.5%)	0.71†
>1.7 mmol/L, n (%)	670 (40.1%)	279 (39.6%)	391 (40.5%)	
Smoking status, n (%)				0.40†
Non-smoker	1118 (68.2%)	455 (69.4%)	663 (67.4%)	
Ex-smoker/smoker	522 (31.8%)	201 (30.6%)	321 (32.6%)	
BMI				
Median (IQR), kg/m^2^	30.8 (27.3–35.1)	30.7 (27.3–35.0)	30.8 (27.1–35.2)	0.62*
<25 kg/m^2^, n (%)	198 (11.6%)	78 (11.0%)	120 (12.0%)	0.72†
25–29.9 kg/m^2^, n (%)	559 (32.6%)	238 (33.5%)	321 (32.0%)	
≥30 kg/m^2^, n (%)	957 (55.8%)	395 (55.6%)	562 (56.0%)	
eGFR				
Median (IQR), mL/min	75.0 (64.0–87.0)	75.0 (65.0–86.0)	75.0 (63.0–87.0)	0.35*
≥90 mL/min, n (%)	337 (19.3%)	138 (19.4%)	199 (19.3%)	0.94†
<90 mL/min, n (%)	1405 (80.7%)	572 (80.6%)	833 (80.7%)	
ACR				
Median (IQR), mg/mmol	1.2 (0.5–2.9)	1.1 (0.5–2.6)	1.2 (0.6–3.1)	0.24*
<3 mg/mmol, n (%)	688 (75.4%)	299 (77.9%)	389 (73.7%)	0.15†
≥3 mg/mmol, n (%)	224 (24.6%)	85 (22.1%)	139 (26.3%)	
AMD status				
No AMD	1703 (81.5%)	730 (87.3%)	973 (77.7%)	<0.001†
Early AMD	284 (13.6%)	78 (9.3%)	206 (16.4%)	<0.001†
Intermediate AMD	90 (4.3%)	24 (2.9%)	66 (5.3%)	0.002†
Any late AMD	12 (0.6%)	4 (0.5%)	8 (0.6%)	0.51†
DR status				1.00†
No DR	1547 (74.3%)	620 (74.3%)	927 (74.3%)	
Background/NPDR	507 (24.4%)	203 (24.3%)	304 (24.4%)	
STDR	27 (1.3%)	11 (1.3%)	16 (1.3%)	

Participants had type 2 diabetes and were aged 50 years and older.

AMD status: no AMD versus any AMD, and no AMD versus each grade individually.

* P value relates to the difference between groups examined using Wilcoxon rank-sum/Mann-Whitney test.

† P value relates to the difference between groups examined using Pearson’s χ2 test.

ACR, albumin creatinine ratio; AMD, age-related macular degeneration; BMI, body mass index; BP, blood pressure; DM, diabetes mellitus; DR, diabetic retinopathy; eGFR, estimated glomerular filtration rate; HbA1c, glycosylated haemoglobin; HDL, high density lipoprotein; LDL, low density lipoprotein; NPDR, non-proliferative diabetic retinopathy; STDR, sight-threatening diabetic retinopathy.

### AMD grading concordance

The inter-rater agreement of AMD grading between the principal grader (DFR) and a senior reading centre grader (DGP) on a random 20% sample was 90.74% for right eyes and 89.07% for left eyes. The kappa statistics were 0.79 and 0.77, respectively (online supplemental tables S1 and S2). This is interpreted as excellent agreement^30^ and supports analysis using a single grader.

### Association of metformin use with

Table 3 shows the logistic regression analysis of association between metformin use during the study period and incidence and progression of AMD. In univariate analysis, metformin use was associated with reduced incidence of intermediate AMD (OR 0.60, 95% CI 0.44 to 0.83, p=0.002) and late AMD (OR 0.43, 95% CI 0.21 to 0.88, p=0.02). After controlling for age and sex, the association between metformin use and incidence of intermediate AMD remained significant (OR 0.65, 95% CI 0.47 to 0.90, p=0.009), but late AMD incidence did not (OR 0.59, 95% CI 0.28 to 1.22, p=0.2). The incident numbers with late AMD were relatively small, 34 no metformin and 10 metformin users.

**Table 3:** Logistic regression analysis between metformin use and incidence of AMD at different stages

Outcome	Record of metformin use	N cases	Unadjusted*		Age and sex adjusted	
			OR (95% CI)	P value	OR (95% CI)	P value
Incidence of early AMD (at risk at BL=1608; no AMD at BL, 1703, −95 with no AMD at BL and intermediate or late AMD at FU)	No (n=914)	108				
	Yes (n=694)	80	0.97 (0.71 to 1.32)	0.9	1.00 (0.73 to 1.36)	0.9
Incidence of intermediate AMD (at risk at BL=1957; no or early AMD at BL, 1987, −30 with no AMD or early AMD at BL and late AMD at FU)	No (n=1155)	132				
	Yes (n=802)	58	0.60 (0.44 to 0.83)	**0.002**	0.65 (0.47 to 0.90)	**0.009**
Incidence of late AMD (at risk at BL=2077, no, early or intermediate AMD at BL)	No (n=1245)	34				
	Yes (n=832)	10	0.43 (0.21 to 0.88)	**0.02**	0.59 (0.28 to 1.22)	0.2
Incidence of any AMD (at risk at BL=1703; no AMD at BL)	No (n=973)	167				
	Yes (n=730)	116	0.91 (0.70 to 1.18)	0.5	0.95 (0.73 to 1.23)	0.7
Progression from early/intermediate to late AMD (at risk at BL=374; early or intermediate AMD at BL)	No (n=272)	29				
	Yes (n=102)	9	0.81 (0.37 to 1.78)	0.6	0.96 (0.42 to 2.19)	0.9

P values <0.05 in bold

* Univariate logistic regression.

AMD, age-related macular degeneration; BL, basline; FU, follow-up.

In the multivariate analysis (table 4), the association between metformin use and incident intermediate AMD persisted after controlling for age, sex, presence of DR, glycaemic control (HbA1c) and diabetes duration (complete record: OR 0.64, 95% CI 0.45 to 0.90, p=0.01). Results were similar after using multiple imputation to missing data (OR 0.66, 95% CI 0.47 to 0.91, p=0.01). Rates of missing data are given in online supplemental table S3. Controlling for high density lipoprotein, baseline AMD and body mass index (variables with a p value <0.2 in the univariate analysis, online supplemental tables S4–S6) did not materially affect these results (incidence of intermediate AMD (OR 0.66, 95% CI 0.48 to 0.91, p=0.01) with multiple imputation). Although smoking is a known risk factor for AMD, its OR significance did not meet a p value <0.2 in univariate analysis and was not included in our multivariate model according to the predetermined criteria.

**Table 4:** Multivariate logistic regression analysis of association between metformin use and AMD

Outcome	Record of metformin use	Adjusted (Model 1)*		Adjusted (Model 2)†	
		OR (95% CI)	P value	OR (95% CI)	P value
Incidence of early AMD	Yes	0.99 (0.71 to 1.37)	0.9	0.98 (0.72 to 1.34)	0.9
Incidence of intermediate AMD	Yes	0.64 (0.45 to 0.90)	**0.01**	0.66 (0.47 to 0.91)	**0.01**
Incidence of late AMD	Yes	1.14 (0.48 to 2.73)	0.8	0.57 (0.26 to 1.23)	0.1
Incidence of any AMD	Yes	0.94 (0.71 to 1.24)	0.7	0.95 (0.72 to 1.25)	0.7
Progression from early/intermediate to late AMD	Yes	1.73 (0.64 to 4.73)	0.3	0.83 (0.35 to 1.96)	0.7

No metformin use was the reference.

P values <0.05 in bold

* Multivariable logistic regression adjusted by baseline age, sex, presence of DR, HbA1c and known diabetes duration (complete record analysis).

† Multivariable logistic regression adjusted by baseline age, sex, presence of DR, HbA1c and known diabetes duration (multiple imputation to missing data).

AMD, age-related macular degeneration; DR, diabetic retinopathy; HbA1c, glycosylated haemoglobin.

No significant association was detected between metformin use and incidence of early AMD, any AMD and progression from early or intermediate to late AMD (p>0.05), including after controlling for age and sex (p>0.05).

## Discussion

Using data from a whole population DR screening programme, we have found that metformin use in people with diabetes was associated with reduced incidence of intermediate AMD over 5 years, which was consistent across regression models. The OR point estimate in the adjusted models indicated metformin was associated with 37% fewer new cases of intermediate AMD over 5 years, with 95% CIs of between 8% and 57% reduction. This result merits further study as reducing the incidence of AMD at an intermediate stage before vision loss would be highly desirable for patients and health systems.

Although we found an association between metformin and reduced progression to late AMD in unadjusted logistic regression, multivariable-adjusted logistic regression did not uphold this association. There was a relatively small number of cases progressing to late AMD which will have affected the power of this study to show a significant effect. A reduced 5year incidence of intermediate AMD associated with metformin use strengthens evidence of an effect by metformin on AMD but at an earlier stage of the disease.

This study graded the AMD of all participants from colour fundus images, which is a strength over studies which have retrospectively used secondary data such as insurance claims^31^,^39^ or primary care records.^40^ AMD diagnosis has been taken from these sources without severity grading or validation of its accuracy. Vergroesen *et al* used colour fundus photographs to identify AMD in the Rotterdam Eye Study. They found metformin use was associated with reduced prevalence of any AMD (OR 0.69, 95% CI 0.49 to 0.98), but not with the incidence of new AMD.^22^ They did not include data on different grades of AMD. We have graded AMD severity according to a modified AREDS grading with quality assurance. We used AREDS AMD Levels 1–4^28^ as clinically relevant, although this may limit comparison to epidemiological studies using the AREDS 9 point AMD severity scale.^41^ AMD has a very wide spectrum from a few drusen of early AMD and low risk of progression to advanced AMD with visual loss, so using an unqualified diagnosis of AMD risks grouping disparate patients together and masking effects.

Metformin has multiple treatment effects potentially targeting early/intermediate AMD and reducing progression to vision-damaging late AMD.^13 17 42 43^ Its antioxidant^8^ and anti-inflammatory^14^ effects are promising for treating age-related diseases, including AMD. A study on a human adult retinal pigment epithelial cell line (adult retinal pigment epithelial (ARPE)-19) and rat models found that metformin could promote autophagy and reduce induced apoptosis of retinal cells.^43^ Autophagy is an essential process for the clearance of aberrant cell components, including by the retinal pigment epithelium, which is reduced in AMD.^43^,^45^ The benefits of these effects are likely to be in the prevention of damaging effects of ageing rather than in their reversal, but further laboratory studies are needed to investigate the potential mechanisms of metformin in AMD.

A recent bioinformatic approach to identify novel therapeutic candidates for AMD by identifying medications that can affect multiple genes involved in AMD identified that metformin had the strongest association with neovascular AMD genes and was among the top candidates for all dry AMD subtypes.^6^ However, a small phase 2 trial of metformin versus observation in GA recruited 66 patients with a mean GA area of 7.45 mm, 85% of whom had foveal-centre involvement.^46^ It found no effect of metformin on the rate of GA growth. No trials have yet been done on earlier stage AMD, but our results would support a clinical trial in earlier stage disease.

Apart from this one small phase 2 trial, the literature on metformin and AMD is confined to observational studies which are subject to inherent biases including our own. Huh *et al* found a significant risk of bias, particularly due to confounding, across all studies.^21^ A significant limitation of our study was that it was observational and there were baseline differences between the group taking metformin and the group who were not. Most notably, there was significantly more AMD at baseline in the group not taking metformin, making progression more likely. We adjusted for baseline differences between the metformin and no metformin groups, including in AMD in our model, although we acknowledge that this is a potential source of bias in our results. The limitations of our study are that we did not have data regarding the dose, duration of prior use and compliance of metformin use. We included patients who were prescribed metformin at baseline and during follow-up within the metformin use group. Including patients on metformin for a short time prior to baseline would potentially reduce the ability of our study to pick up an association with AMD changes and reduce the size of any association. The group not prescribed metformin was older both in median age and proportion than the older age groups. Again, this will have reduced the ability of our study to identify an association with AMD, and an association with reduced incident intermediate AMD over 5 years was still found.

Our study had relatively small numbers developing advanced AMD, which is inevitable with a population-based study. We did not have data on diet or AREDS supplement use as this is not kept on GP records from which our data were extracted, and so we were unable to include this in our model. Smoking was not included in our model because it did not meet predetermined criteria in the univariate analysis. This may have affected our dataset which combined ex-smokers and current smokers in a single category versus non-smokers, and the degree of missing data for this item (21.7%).

We acknowledge that optical coherence tomography would have better sensitivity at detecting AMD than colour fundus photographs; however, it was not available for this cohort. Finally, our results are applicable to people with diabetes. They were the subject of this study as they are the only large group currently prescribed metformin, having regular fundus imaging as a whole population. The generalisability of these findings to the general population is unknown.

Our study, using validated AMD grading, has identified an association between oral metformin use and reduced risk of developing intermediate AMD over 5 years. Given metformin’s potential favourable effects on AMD’s pathophysiology, this reduction in risk seems biologically plausible. There remains a lack of data from prospective clinical studies of metformin in AMD. Our study supports undertaking a prospective clinical trial of metformin for reducing progression of AMD prior to the onset of advanced AMD.

## Supplementary material

10.1136/bmjophth-2025-002339 online supplemental file 1

## Data Availability

Data are available in a public, open access repository.
