# Supplementary material for: Metformin and incidence of age-related macular degeneration in people with diabetes: a population-based 5-year case-control study
Source: BMJ Open Ophthalmol. 2026 Feb 2;11(1):e002339. doi: 10.1136/bmjophth-2025-002339 (PMC12878204; doi:10.1136/bmjophth-2025-002339)
Supplement: online supplemental file 1 [file bmjophth-11-1-s001.docx]

Supplementary data

Table S1 Inter-rater agreement table for right eyes.

| Right eye | | 2nd grader | | | | | |
| --- | --- | --- | --- | --- | --- | --- | --- |
|  |  | No AMD | Early | Intermediate | Late | Ungradable | Total |
| 1st grader | No AMD | 288 | 3 | 7 | 0 | 1 | 299 |
|  | Early | 11 | 55 | 6 | 0 | 0 | 72 |
|  | Intermediate | 1 | 7 | 32 | 1 | 0 | 41 |
|  | Late | 0 | 0 | 0 | 6 | 0 | 6 |
|  | Ungradable | 1 | 0 | 0 | 1 | 1 | 3 |
|  | Total | 301 | 65 | 45 | 8 | 2 | 421 |

Inter-rater statistic of right eye.

| Right eye | | | |
| --- | --- | --- | --- |
| Agreement | Expected agreement | Kappa | Standard error |
| **90.74%** | **54.49%** | **0.79** | **0.03** |

Table S2 Inter-rater agreement table for left eyes.

| Left eye | | 2nd grader | | | | | |
| --- | --- | --- | --- | --- | --- | --- | --- |
|  |  | No AMD | Early | Intermediate | Late | Ungradable | Total |
| 1st grader | No AMD | 275 | 6 | 3 | 0 | 2 | 286 |
|  | Early | 14 | 52 | 8 | 0 | 1 | 75 |
|  | Intermediate | 2 | 8 | 38 | 0 | 0 | 48 |
|  | Late | 0 | 0 | 2 | 7 | 0 | 9 |
|  | Ungradable | 0 | 0 | 0 | 0 | 3 | 3 |
|  | Total | 291 | 66 | 51 | 7 | 6 | 421 |

Inter-rater statistic of left eye.

| Left eye | | | |
| --- | --- | --- | --- |
| Agreement | Expected agreement | Kappa | Standard error |
| **89.07%** | **51.18%** | **0.77** | **0.03** |

| **Variable** | **Missing** |
| --- | --- |
|  | **(%)** |
| Age | 0 (0%) |
| Sex, n (%) | 0 (0%) |
| Ethnicity, n (%) | 356 (13.8%) |
| DM type, n (%) | 157 (6.1%) |
| HbA1c | 326 (12.6%) |
| Known DM duration | 0 (0%) |
| Diastolic BP | 239 (9.2%) |
| Systolic BP | 238 (9.2%) |
| Total cholesterol | 423 (16.3%) |
| HDL | 446 (17.2%) |
| LDL | 521 (20.1%) |
| Triglycerides | 541 (20.9%) |
| Smoking status | 563 (21.7%) |
| BMI | 470 (18.2%) |
| eGFR | 440 (17.0%) |
| ACR | 1429 (55.2%) |
| Abbreviations: ACR, albumin creatinine ratio; AMD, age-related macular degeneration; BMI, body mass index; BP, blood pressure; DM, diabetes mellitus; DR, diabetic retinopathy; eGFR, estimated glomerular filtration rate; HDL, high density lipoprotein; IQR, inter quartile range; LDL, low density lipoprotein. | |

**Table S3. Degree of missingness for individual variables**

**Table S4. Logistic regression of risk factors for 5-year incidence of early AMD in PWD**

| Risk factors | Unadjusted | |
| --- | --- | --- |
|  | **OR (95% CI)** | **P** |
| Presence of DR |  |  |
| No DR | reference |  |
| Any DR | 1.32 (0.95-1.83) | **0.1*** |
| Age (per increase 1 year) | 1.02 (1.00-1.04) | **0.06*** |
| Sex |  |  |
| Male | reference |  |
| Female | 1.11 (0.82-1.50) | 0.5 |
| HbA1c (per increase 1 mmol/mol) | 1.00 (0.99-1.01) | 0.5 |
| Known DM duration (per increase 1 year) | 1.01 (0.99-1.03) | 0.3 |
| Diastolic pressure (per increase 1 mm Hg) | 0.99 (0.98-1.01) | 0.5 |
| Systolic pressure (per increase 1 mm Hg) | 1.00 (0.99-1.02) | 0.4 |
| Total cholesterol (per increase 1 mmol/l) | 0.87 (0.74-1.03) | **0.1*** |
| HDL (per increase 1 mmol/l) | 1.66 (1.07-2.57) | **0.03*** |
| LDL (per increase 1 mmol/l) | 0.84 (0.69-1.03) | **0.09*** |
| Triglycerides (per increase 1 mmol/l) | 0.86 (0.72-1.03) | **0.1*** |
| Smoking |  |  |
| Non-smoker | reference |  |
| Ex-smoker/smoker | 0.77 (0.53-1.12) | 0.2 |
| BMI (per increase 1 kg/m^2^) | 0.99 (0.96-1.01) | 0.4 |
| eGFR (per increase 1 ml/min) | 1.00 (0.99-1.01) | 0.6 |

Abbreviations: AMD, age-related macular degeneration; BMI, body mass index; DM, diabetes mellitus; DR, diabetic retinopathy; eGFR, estimated glomerular filtration rate; HDL, high density lipoprotein; LDL, low density lipoprotein; OR, odds ratio; PWD, people with diabetes.

**Table S5.** **Logistic regression of risk factors for 5-year incidence of intermediate AMD in PWD.**

| Risk factors | Unadjusted | |
| --- | --- | --- |
|  | **OR (95% CI)** | **P** |
| Presence of DR |  |  |
| No DR | reference |  |
| Any DR | 0.99 (0.70-1.39) | 0.9 |
| Age (per increase 1 year) | 1.06 (1.04-1.07) | **<0.001*** |
| Sex |  |  |
| Male | reference |  |
| Female | 1.24 (0.92-1.67) | 0.2 |
| HbA1c (per increase 1 mmol/mol) | 1.00 (0.98-1.01) | 0.4 |
| Known DM duration (per increase 1 year) | 1.01 (0.98-1.03) | 0.5 |
| Diastolic pressure (per increase 1 mm Hg) | 0.99 (0.97-1.01) | 0.2 |
| Systolic pressure (per increase 1 mm Hg) | 1.00 (0.99-1.01) | 0.4 |
| Total cholesterol (per increase 1 mmol/l) | 0.94 (0.80-1.10) | 0.4 |
| HDL (per increase 1 mmol/l) | 1.60 (1.04-2.45) | **0.03*** |
| LDL (per increase 1 mmol/l) | 0.88 (0.72-1.08) | 0.2 |
| Triglycerides (per increase 1 mmol/l) | 0.90 (0.76-1.07) | 0.2 |
| Smoking |  |  |
| Non-smoker | reference |  |
| Ex-smoker/smoker | 0.82 (0.57-1.19) | 0.3 |
| BMI (per increase 1 kg/m^2^) | 0.98 (0.95-1.00) | **0.09*** |
| eGFR (per increase 1 ml/min) | 1.00 (0.99-1.01) | 0.9 |

Abbreviations: AMD, age-related macular degeneration; BMI, body mass index; DM, diabetes mellitus; DR, diabetic retinopathy; eGFR, estimated glomerular filtration rate; HDL, high density lipoprotein; LDL, low density lipoprotein; OR, odds ratio; PWD, people with diabetes.

**Table S6. Logistic regression of risk factors for 5-year incidence of late AMD in PWD**

| Risk factors | Unadjusted | |
| --- | --- | --- |
|  | **OR (95% CI)** | **P** |
| Presence of DR |  |  |
| No DR | reference |  |
| Any DR | 1.32 (0.70-2.51) | 0.4 |
| Age (per increase 1 year) | 1.17 (1.13-1.22) | **<0.001*** |
| Sex |  |  |
| Male | reference |  |
| Female | 1.48 (0.82-2.67) | 0.2 |
| HbA1c (per increase 1 mmol/mol) | 0.99 (0.97-1.02) | 0.6 |
| Known DM duration (per increase 1 year) | 0.97 (0.90-1.05) | 0.5 |
| Diastolic pressure (per increase 1 mm Hg) | 0.96 (0.93-0.99) | **0.02*** |
| Systolic pressure (per increase 1 mm Hg) | 0.99 (0.96-1.01) | 0.2 |
| Total cholesterol (per increase 1 mmol/l) | 1.00 (0.72-1.39) | 0.9 |
| HDL (per increase 1 mmol/l) | 1.65 (0.68-3.97) | 0.3 |
| LDL (per increase 1 mmol/l) | 1.01 (0.68-1.50) | 0.9 |
| Triglycerides (per increase 1 mmol/l) | 0.82 (0.54-1.23) | 0.3 |
| Smoking |  |  |
| Non-smoker |  |  |
| Ex-smoker/smoker | 1.23 (0.60-2.52) | 0.5 |
| BMI (per increase 1 kg/m^2^) | 0.92 (0.86-0.99) | **0.02*** |
| eGFR (per increase 1 ml/min) | 0.96 (0.94-0.98) | **<0.001*** |

Abbreviations: AMD, age-related macular degeneration; BMI, body mass index; DM, diabetes mellitus; DR, diabetic retinopathy; eGFR, estimated glomerular filtration rate; HDL, high density lipoprotein; LDL, low density lipoprotein; OR, odds ratio; PWD, people with diabetes.
